# Total Hip Arthroplasty Patients With Systemic Sclerosis Have Worse Medical Outcomes But Clinically Similar Implant Survival Independent of Immunomodulatory Therapy

**DOI:** 10.5435/JAAOSGlobal-D-24-00257

**Published:** 2025-04-04

**Authors:** Anthony E. Seddio, Helia Hosseini, Rajiv S. Vasudevan, Michael J. Gouzoulis, Lee E. Rubin, Jonathan N. Grauer

**Affiliations:** From the Department of Orthopaedics and Rehabilitation, Yale School of Medicine, New Haven, CT.

## Abstract

**Introduction:**

Systemic sclerosis (SSc) is a multisystem autoimmune disorder characterized by fibrosis and often early articular degeneration. Total hip arthroplasty (THA) is a procedure for which SSc patients may be considered. However, outcomes past hospital discharge and the association of exposure to common immunomodulatory therapy (IMT) agents on such outcomes remain unknown.

**Methods:**

Retrospective cohort study of SSc patients who underwent THA. Patients with SSc were matched in 4:1 ratio with (−)SSc controls based on age, sex, and Elixhauser Comorbidity Index. Incidence of 90-day medical and implant-related adverse events (AEs) were assessed by multivariable logistic regression, and 5-year revision was assessed by Kaplan-Meier survival analysis and log-rank test.

**Results:**

SSc patients undergoing THA demonstrated greater odds ratio (OR) of severe (OR 1.46) and minor AEs (OR 1.47; *P* < 0.001 for both). However, perioperative IMT utilization was not associated with notable modification of these odds (*P* > 0.05 for both). SSc patients demonstrated similar odds of 90-day implant-related AEs (*P* > 0.05 for all) and similar 5-year revision-free survival vs (−)SSc controls (97.3% vs. 96.6%, respectively; *P* = 0.200).

**Discussion:**

Patients with SSc undergoing THA experience increased 90-day medical AEs, independent of IMT utilization. Encouragingly, SSc patients demonstrated similar 90-day implant-related AEs and 5-year revision-free survival, suggesting that the major barrier to superior outcomes may not be implant related but rather driven by medical complications.

Systemic sclerosis (SSc), also known as scleroderma, is a multisystem autoimmune disorder primarily involving the skin, internal organs, and musculoskeletal (MSK) system.^1-3^ Despite being relatively rare, affecting two per 10,000 patients, it is associated with exceedingly high morbidity and has the highest case-specific mortality of any rheumatic disease (RD).^4^ MSK manifestations of synovitis, arthralgia, and joint contracture are common among those with SSc,^5^ with articular involvement seen in 46% to 95% of patients.^6^ These manifestations may necessitate intervention to address advanced joint degeneration, such as total hip arthroplasty (THA).

Although THA is known to be a very successful surgery, patients with underlying RD have demonstrated an elevated risk of infectious postoperative complications,^7^ potentially amplified by frequent immunomodulatory therapy (IMT) utilization.^8^ In addition, implant-related adverse events (AEs), such as prosthetic dislocation, may be elevated in these patients due to abnormal soft-tissue architecture.^9-11^ A study by Singh and Cleveland used the National Inpatient Sample to assess patients with SSc undergoing THA, reporting heightened in-hospital odds of transfusion, longer length of stay, nonhome discharge, and a staggering nine times greater odds of revision during the index admission.^12^ However, it remains unknown if these outcomes translate to the postdischarge period, which is critically important to more comprehensively characterize THA outcomes in this patient population.

Like many autoimmune conditions, SSc is frequently managed with various IMT agents.^13^ Within the first year following diagnosis, one-third of all SSc patients begin IMT.^14^ Although these therapeutic agents may provide patients a greater degree of symptom control, they have demonstrated an independent association with heightened risks of medical and surgical complications, such as infection, sepsis, and poor wound healing.^8,15,16^ These associations have catalyzed clinical guidelines for perioperative optimization of IMT for patients with various RDs undergoing total joint arthroplasty (TJA).^17^ Despite these robust clinical guidelines, SSc has not been specifically addressed, nor have prior studies on patients with SSc considered the association of IMT on postoperative outcomes.

The paucity of literature investigating orthopaedic surgery outcomes in patients with SSc and the lack of inclusion within perioperative IMT optimization guidelines underscores the vulnerability of these patients. Based on these knowledge gaps, this study leveraged a large, national, multi-insurance administrative data set to identify a robust cohort of patients with SSc undergoing THA. We hypothesized that SSc would be associated with inferior postoperative outcomes and that preoperative IMT utilization would amplify this association.

## Methods

### Database and Study Cohort

Data were extracted from the 2010 to Q1 2023 PearlDiver Mariner 170 database (PearlDiver Technologies). This national, multi-insurance, administrative database contains records on nearly 170 million patients. The use of this database has been well established in the orthopaedic literature.^18-21^ As data from this database are deidentified, our institutional review board found studies using this database exempt from review.

All adult patients diagnosed with hip osteoarthritis undergoing primary THA were identified using International Classification of Disease (ICD) codes and the Current Procedural Terminology (CPT) code 27130, respectively. Patients were excluded from the study if they were younger than18 years, had less than 90 days of follow-up after surgery, or presented for trauma, neoplasm, or infection. Demographic data abstracted included age, sex, and Elixhauser Comorbidity Index (ECI, a measure of overall comorbidity burden).

**Figure 1:**
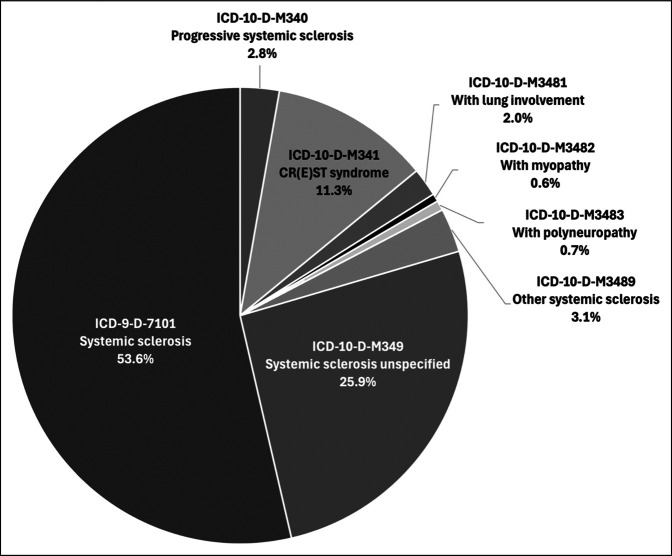
Pie chart reflecting the distribution of systemic sclerosis patients undergoing total hip arthroplasty (THA) based on International Classification of Disease (ICD) codes.

Patients diagnosed with SSc before surgery were identified based on ICD codes (Supplementary Table 1, http://links.lww.com/JG9/A400). These codes have been previously validated, demonstrating a positive predictive value of 93%.^22^ The distribution of SSc patients undergoing THA based on these ICD diagnostic codes is shown (Figure 1).

THA patients with SSc were matched in 1:4 ratio to (−)SSc controls based on age, sex, and ECI to account for potential differences in underlying patient demographics and comorbidity burden. Importantly, matching on ECI considers the presence of the following comorbidities: congestive heart failure, cardiac arrhythmias, valvular disease, pulmonary circulation disorders, peripheral vascular disorders, hypertension, paralysis, other neurological disorders, chronic pulmonary disease, diabetes, hypothyroidism, renal failure, liver disease, peptic ulcer disease, AIDS/HIV, lymphoma, solid tumor without metastasis, rheumatoid arthritis/collagen vascular diseases, coagulopathy, obesity (body mass index >30), weight loss, fluid/electrolyte disorders, blood loss anemia, deficiency anemia, alcohol abuse, drug abuse, psychoses, and depression.

### Ninety-Day Adverse Events and Five-Year Revision-Free Survival

Ninety-day postoperative AEs were determined based on ICD codes. These AEs were individually reported and aggregated by complication severity. Severe medical AEs (SMAE) included sepsis, cardiac event (myocardial infarction or cardiac arrest), deep vein thrombosis (DVT), pulmonary embolism, and surgical site infection (SSI), which included both superficial SSI and deep SSI. Minor medical AEs (MMAE) included pneumonia, urinary tract infection (UTI), wound dehiscence, hematoma, acute kidney injury, and transfusion. Any medical AE (AMAE) was noted when there was the occurrence of a severe or minor AE.

Ninety-day implant-related AEs were also determined, including incidence of prosthetic dislocation, periprosthetic fracture, and aseptic loosening. Emergency department (ED) visits within 90 days were determined based on the occurrence of corresponding ED-specific evaluation and management codes. The occurrences of hospital readmissions within the 90-day postoperative period were identified using the PearlDiver “*admission*” function that identifies relevant inpatient ICD codes.

To determine revision-free survival, the occurrence of THA revision was determined by tracking occurrence of the CPT codes 27134, 27137, or 27138 within the 5 years following primary THA.

### Immunomodulatory Therapy Utilization

For subanalyses, patients using IMT (SSc [+]IMT) within 1 year before their THA and patients not using IMT (SSc [−]IMT) were determined. Agents assessed as IMT included methotrexate, mycophenolate mofetil, cyclophosphamide, nintedanib, and tocilizumab. These IMT agents were identified based on prescription billing records using the National Drug Code associated with each within 1 year before surgery. These IMT agents are all United States Food and Drug Administration approved as therapeutics for the management of SSc.^23-26^ Glucocorticoid agents, often used in other RDs, are contraindicated in patients with SSc due to their association with precipitating scleroderma renal crisis^27^ and therefore excluded.

### Data Analyses

Patient characteristics, including mean age, sex, and ECI of patients with and without SSc who underwent THA, were tabulated and compared using the independent two-tailed student *t*-test or Pearson χ^2^ test, where appropriate, for the unmatched and matched populations.

Univariable comparisons of postoperative AEs between matched patients with and without SSc were done using Pearson χ^2^ test. Multivariable logistic regressions of postoperative AEs were then done controlling for age, sex, and ECI. Odds ratio (OR) and 95% confidence intervals (CIs) for each independent association were determined.

The 5-year revision free survival was determined by rates of THA revision using a Kaplan-Meier survival analysis. The log-rank (Mantel-Cox) test was used to statistically analyze the rates of revision between patients with SSc versus (−)SSc matched controls.

For IMT sub analyses, the OR and 95% CI of AMAE, SMAE, or MMAE was first determined for SSc (−)IMT and SSc (+)IMT compared with (−)SSc-matched controls undergoing THA. Then, a subsequent multivariable logistic regression directly comparing SSc (−)IMT vs. SSc (+)IMT patients was done to determine whether the odds of AMAE, SMAE, or MMAE were statistically different between SSc patients with or without perioperative IMT use.

All statistical tests were done using the RStudio package (Posit) embedded within the PearlDiver Bellwether software. Tables were constructed using Microsoft Excel (Microsoft Corporation) and figures using Prism 10 (GraphPad Software).

## Results

### Study Cohort

In total, 748,671 patients without SSc undergoing THA and 1,234 patients with SSc undergoing THA were identified (Table 1). In the unmatched groups, those with SSc were younger, more frequently female, and higher ECI (*P* < 0.001 for all).

**Table 1:** Unmatched and Matched Patient Demographics of Those With and Without Systemic Sclerosis Who Underwent Total Hip Arthroplasty

Factor or Variable	THA (−)SSc (n = 748,671)	THA (+)SSc (n = 1234)	*P*	Matched THA (−)SSc (n = 4841)	Matched THA (+)SSc (n = 1217)	*P*
Age ± SD	63.8 ± 10.0	59.3 ± 11.4	**<0.001**	59.8 ± 10.7	59.8 ± 10.8	0.795
Sex (%)			**<0.001**			1.000
Female	420,946 (56.2%)	1051 (85.2%)		4112 (85.0%)	1034 (85.0%)	
Male	327,725 (43.8%)	183 (14.8%)		729 (15.0%)	183 (15.0%)	
ECI ± SD	4.3 ± 3.3	7.3 ± 3.9	**<0.001**	7.1 ± 3.8	7.2 ± 3.9	0.732

ECI = Elixhauser Comorbidity Index; SSc = systemic sclerosis; THA = total hip arthroplasty

Bolding indicates significance, *P* < 0.05.

After matching, 4841 patients without SSc and 1217 patients with SSc were there. In these matched groups, there were no longer differences in age, sex, and ECI (*P* > 0.05 for all).

### Outcomes of Total Hip Arthroplasty Patients With Versus Without Systemic Sclerosis

The results of our univariable and multivariable analysis of 90-day outcomes following THA in matched patients with and without SSc are shown (Table 2). By the univariable analysis, THA (+)SSc patients exhibited a markedly higher incidence of a number of individual medical AEs, ED visits, and aggregated AMAE, SMAE, and MMAE (*P* < 0.05 for all). Conversely, implant-related AEs (including prosthetic dislocation, aseptic loosening, and periprosthetic fracture) occurred at similar rates for THA (+)SSc patients compared with THA (−)SSc patients (*P* > 0.05 for all).

**Table 2:** Univariable and Multivariable Analysis of Adverse Events Within 90 Days of Total Hip Arthroplasty

Factor or Variable	Matched THA (−)SSc (n = 4841)	Matched THA (+)SSc (n = 1217)	*P*	OR (95% CI)	*P*
Any medical adverse event	1404 (29.0)	443 (36.4)	**<0.001**	1.43 (1.24-1.64)	**<0.001**
Severe medical adverse event	502 (10.4)	174 (14.3)	**<0.001**	1.46 (1.20-1.76)	**<0.001**
Sepsis	95 (2.0)	56 (4.6)	**<0.001**	2.44 (1.72-3.43)	**<0.001**
Cardiac event	71 (1.5)	27 (2.2)	0.083	1.51 (0.95-2.34)	0.074
DVT	197 (4.1)	68 (5.6)	**0.025**	1.39 (1.03-1.84)	**0.025**
SSI	135 (2.8)	45 (3.7)	0.115	1.33 (0.93-1.86)	0.108
PE	115 (2.4)	34 (2.8)	0.460	1.17 (0.78-1.71)	0.441
Minor medical adverse event	1160 (24.0)	381 (31.3)	**<0.001**	1.47 (1.27-1.69)	**<0.001**
Pneumonia	191 (3.9)	92 (7.6)	**<0.001**	2.02 (1.55-2.63)	**<0.001**
UTI	614 (12.7)	221 (18.2)	**<0.001**	1.54 (1.30-1.83)	**<0.001**
Wound dehiscence	105 (2.2)	38 (3.1)	0.064	1.44 (0.98-2.09)	0.057
Hematoma	80 (1.7)	21 (1.7)	0.958	1.04 (0.62-1.65)	0.880
AKI	288 (5.9)	74 (6.1)	0.916	1.00 (0.76-1.31)	0.977
Transfusion	253 (5.2)	59 (4.8)	0.645	0.92 (0.68-1.22)	0.569
Implant-related adverse event	125 (2.6)	42 (3.5)	0.115	0.89 (0.60-1.29)	0.543
Prosthetic dislocation	78 (1.6)	30 (2.5)	0.056	0.98 (0.61-1.55)	0.950
Periprosthetic fracture	42 (0.9)	12 (1.0)	0.815	0.79 (0.39-1.54)	0.521
Aseptic loosening	21 (0.4)	<11	0.799	0.41 (0.12-1.14)	0.119
ED visit	1205 (24.9)	380 (31.2)	**<0.001**	1.40 (1.21-1.62)	**<0.001**
Readmission	472 (9.8)	119 (9.8)	1.000	1.00 (0.80-1.23)	0.977

AKI = acute kidney injury; CI = confidence interval; DVT = deep vein thrombosis; ED = emergency department; OR = odds ratio; PE = pulmonary embolism; SSc = systemic sclerosis; SSI = surgical site infection; THA = total hip arthroplasty; UTI = urinary tract infection

Bolding indicates significance, *P* < 0.05.

**Figure 2:**
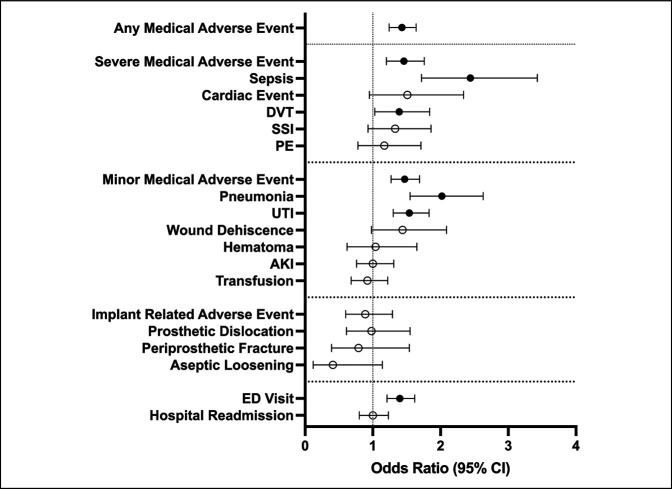
Forest plot showing the odds ratio from the multivariable analysis of 90-day adverse events following total hip arthroplasty (THA) in systemic sclerosis (SSc) patients compared with (−)SSc matched controls. Unfilled circles denote nonsignificant odds ratios. Filled black circles denote notable odds ratios.

Multivariable analysis was then done to assess independent association with AEs (Table 2 and Figure 2). Those with SSc were at greater odds of a number of individual AEs (including sepsis, DVT, pneumonia, UTI, and ED visits) and aggregated AMAE, SMAE, and MMAE (*P* < 0.05 for all). By contrast, no notable associations were found with cardiac events, SSI, pulmonary embolism, wound dehiscence, hematoma, acute kidney injury, or transfusion (*P* > 0.05 for all). Implant-related AEs also revealed no association with SSc (*P* > 0.05 for all). Healthcare utilization metrics revealed that those with SSc have a markedly greater odds of ED visits (*P* < 0.05) but not hospital readmissions (*P* = 0.977).

**Figure 3:**
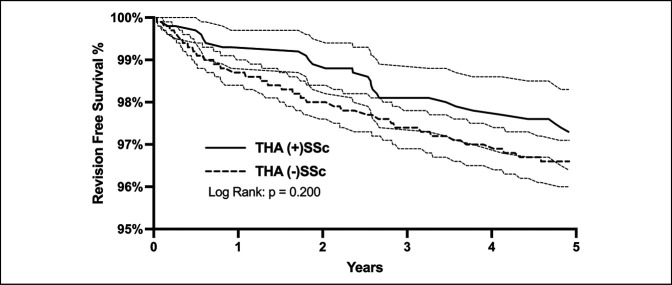
Kaplan-Meier curve showing 5-year revision-free survival in total hip arthroplasty (THA) patients with systemic sclerosis (SSc) versus (−)SSc matched controls.

Kaplan-Meier curve for the 5-year revision-free survival is shown (Figure 3). The rates of revision for THA in (+)SSc vs. (−)SSc patients were found to be similar (97.3% vs. 96.6%, respectively; *P* = 0.200).

### Subanalysis of Those With Versus Without Immunomodulatory Therapy Exposure

The characteristics of SSc patients with versus without IMT exposure within 1 year before THA (301 vs. 949 patients, respectively) are shown (Table 3). Those with IMT exposure were younger and more frequently female than those without (*P* < 0.05 for both). Patients with and without IMT exposure demonstrated similar comorbidity burden (*P* = 0.980).

**Table 3:** Characteristics of Patients With Systemic Sclerosis Who Underwent Total Hip Arthroplasty Who did Not Take Immunomodulatory Medications Within 1 Year Before Surgery Versus Those Who did

Factor or Variable	SSc (−)IMT (n = 949)	SSc (+)IMT (n = 302)	*P*
Age ± SD	60.98 ± 10.41	56.22 ± 12.39	**<0.001**
Sex (%)			**0.002**
Female	780 (82.2%)	271 (89.7%)	
Male	169 (17.8%)	31 (10.3%)	
ECI ± SD	6.97 ± 3.75	6.97 ± 4.05	0.980

ECI = Elixhauser Comorbidity Index; IMT = immunomodulatory therapy; SSc = systemic sclerosis; THA = total hip arthroplasty

Bolding indicates significance, *P* < 0.05

Multivariable analysis was then done for SSc patients with and without IMT exposure relative to (−)SSc controls (Table 4). Compared with (−)SSc controls, both SSc (+)IMT and SSc (−)IMT patients were found to have higher odds of experiencing AMAE, SMAE, and MMAE (*P* < 0.05 for all).

**Table 4:** Multivariable Analysis of 90-Day Adverse Events Following Total Hip Arthroplasty in SSc Patients Who did and did Not Use Immunomodulatory Therapy Within 1 Year Before Surgery Relative to Nonsystemic Sclerosis Total Hip Arthroplasty Control Patients

Factor or Variable	OR (95% CI)	*P*
SSc(−)IMT patients		
Any medical adverse event	1.35 (1.16-1.58)	**<0.001**
Severe medical adverse event	1.31 (1.05-1.63)	**0.014**
Minor medical adverse event	1.42 (1.21-1.66)	**<0.001**
SSc(+)IMT patients		
Any medical adverse event	1.65 (1.26-2.16)	**<0.001**
Severe medical adverse event	2.07 (1.46-2.86)	**<0.001**
Minor medical adverse event	1.56 (1.18-2.07)	**0.002**

CI = confidence interval; IMT = immunomodulatory therapy; OR = odds ratio; SSc = systemic sclerosis; THA = total hip arthroplasty

Bolding indicates significance, *P* < 0.05.

**Figure 4:**
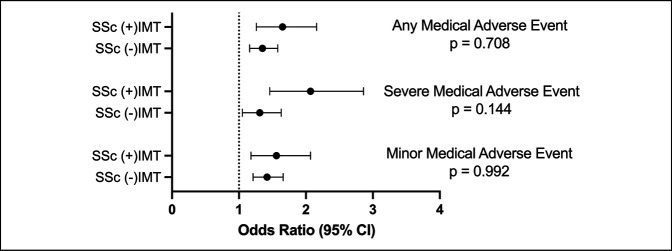
Forest plot showing the odds ratio from the multivariable analysis of 90-day medical adverse events following total hip arthroplasty (THA) in patients who did and did not use immunomodulatory therapy (IMT) preoperatively relative to (−)SSc controls. Overlapping confidence intervals between systemic sclerosis (SSc) (+)IMT vs. SSc (−)IMT reflect nonsignificant differences in these odds (*P* > 0.05 for all).

However, on subsequent multivariable analysis directly comparing the independent ORs of 90-day AEs between SSc (−)IMT vs. SSc (+)IMT patients (Figure 4), no statistically significant difference in odds of AMAE, SMAE, or MMAE were seen, as visually evident by the overlapping 95% CIs (*P* > 0.05 for all).

## Discussion

Often early in the disease, SSc affects the MSK system, which markedly contributes to high patient morbidity.^1-3^ As with other multisystem autoimmune diseases, SSc disease manifestations exist on a spectrum, but the development of advanced articular degeneration has been consistently reported in the literature.^6,28^ The pathophysiology of this articular involvement is progressive, with tethering and joint contracture often leading to impairment in function, thereby leading patients to seek treatment, often in the form of TJA.^12,29^

Prior literature exploring this disease in patients who undergo THA has been limited to in-hospital outcomes and not considered commonly used IMT agents.^12^ As such, this study expands on prior literature by revealing that SSc patients undergoing THA demonstrated greater odds of various 90-day medical complications, but similar implant-related AEs and similar 5-year revision free-survival relative to (−)SSc-matched controls. In addition, this study found that perioperative IMT exposure was not associated with modification of the odds of medical AEs. Encouragingly, these findings refuted our hypothesis, suggesting that patients with SSc undergoing THA do not have a universally elevated risk of complications, and perioperative IMT use is not associated with inferior outcomes.

Our finding that SSc patients were at greater odds of a number of individual 90-day medical complications aligns with the known medical issues associated with SSc.^14^ From an infection perspective, this study found patients with SSc undergoing THA to be at higher odds of sepsis, pneumonia, and UTI. Although the risk of infection following TJA for patients with various RDs remains mixed,^30-32^ SSc patients undergoing total knee arthroplasty have demonstrated a similarly elevated risks of sepsis, pneumonia, and UTI to this study.^33^

This study found patients with SSc trended toward greater odds of cardiac events. Literature has revealed that SSc is an independent risk factor for myocardial infarction with clinical cardiac involvement observed in 10% to 30% of SSc patients.^34^ This study revealed that those with SSc were at an increased odds of DVT, consistent with a known predisposition to hypercoagulation.^35^ Postoperative wound-related complications were encouragingly similar in this study, despite the chronic inflammatory state and tissue fibrosis frequently seen in SSc.^36^ Together, the elevated odds of various medical AEs likely predisposed those with SSc to the increased odds of postoperative emergency service utilization following THA.

The 5-year revision-free survival rate of SSc patients was similar to matched controls without SSc. This finding was reassuring and inconsistent with the prior study using National Inpatient Sample data,^12^ which revealed that SSc patients were at nine times greater odds of in-hospital revision following THA, further highlighting the importance of characterizing outcomes beyond hospital discharge. Orthopaedic surgeons may interpret these similar 5-year revision rates in conjunction with the similar 90-day implant-related complications to reflect that SSc patients may be clinically similar to non-SSc patients regarding long-term implant survival.

Encouragingly, preoperative IMT utilization was not found to amplify the association of postoperative medical complications. Because 90-day aggreged AEs were found to be similar for SSc patients regardless of IMT exposure, this may suggest that SSc is itself the primary driver of the increased odds of medical complications. This finding should be considered within multidisciplinary conversations regarding maintenance of IMT therapy regimens in the perioperative period, which has been recommended in prior literature of other autoimmune RDs.^8^

There are limitations to this study. As with any retrospective administrative database study, it is limited by the coded data used. Because surgical approach cannot be identified through CPT codes, the outcomes between THA approaches should be considered in future studies, as soft-tissue architecture is a key component to the manifestations of this disease and may direct surgical planning. In addition, because therapeutic development for patients with SSc is an area of active research, it is important for future studies to consider the association of other novel therapeutic agents on surgical outcomes.

## Conclusion

In summary, SSc patients undergoing THA experienced greater odds of various 90-day medical complications but similar implant-related AEs and similar 5-year revision-free survival relative to (−)SSc-matched controls. These findings suggest that the major barrier to superior outcomes may not be implant related but rather driven by greater odds of medical complications common to this vulnerable patient population.

## Supplementary Material
