# Supplementary material for: Total Hip Arthroplasty Patients With Systemic Sclerosis Have Worse Medical Outcomes But Clinically Similar Implant Survival Independent of Immunomodulatory Therapy
Source: J Am Acad Orthop Surg Glob Res Rev. 2025 Apr 4;9(4):e24.00257. doi: 10.5435/JAAOSGlobal-D-24-00257 (PMC11975401; doi:10.5435/JAAOSGlobal-D-24-00257)
Supplement: Supplementary file 1 [file jagrr-9-e24.00257-s001.docx]

**Supplementary Table 1.**

Code ICD-10-D-M340 Progressive systemic sclerosis

Code ICD-10-D-M341 CR(E)ST syndrome

Code ICD-10-D-M3481 Systemic sclerosis with lung involvement

Code ICD-10-D-M3482 Systemic sclerosis with myopathy

Code ICD-10-D-M3483 Systemic sclerosis with polyneuropathy

Code ICD-10-D-M3489 Other systemic sclerosis

Code ICD-10-D-M349 Systemic sclerosis unspecified

Code ICD-9-D-7101 Systemic sclerosis
